# Highlight report: Monitoring cytochrome P450 activities in living hepatocytes

**DOI:** 10.17179/excli2017-1039

**Published:** 2017-12-22

**Authors:** Ahmed Ghallab

**Affiliations:** 1 Forensic Medicine and Toxicology Department, Faculty of Veterinary Medicine, South Valley University, Qena, Egypt

## ⁯

Recently, Jannick Theobald and Xinlai Cheng from Heidelberg University published a methods' paper how to monitor cytochrome P450 (CYP) activities in living hepatocytes (Theobald et al., 2017[15]). For this purpose, the authors used substrates that are metabolized by CYP enzymes thereby forming highly fluorescent leaving groups that were quantified by a plate reader. This technique allows repeated real-time measurements of cultivated hepatocytes over extincted time periods (Theobald et al., 2017[15]). The monitoring technique was validated by the use of CYP inducers and was applied to characterize differentiating HepaRG cells. The authors conclude that the fluorescence-based assay can easily be used as a tool to characterize hepatocyte *in vitro* systems.

Hepatotoxicity still represents a major challenge in drug development (Leist et al., 2017[8]; Schenk et al., 2017[12]; Reif et al., 2017[11]; Jansen et al., 2017[6]; Crespo Yanguas et al., 2016[3]; Stöber, 2015[13], 2016[14]; Yanguas et al., 2016[19]; Braeuning and Schwarz, 2016[2]). 

Currently, much effort is invested to develop improved hepatocyte *in vitro* systems (Godoy et al., 2013[5]; Ramboer et al., 2015[10]; Verhulst et al., 2015[18]; Pfeiffer et al., 2015[9]; Kim et al., 2015[7]) and *in silico* techniques (Ghallab et al., 2016[4]; Bartl et al., 2015[1]; Vartak et al., 2016[17]; Thiel et al., 2015[16]). The technique presented by Theobald and colleagues can easily be integrated into *in vitro* systems and should therefore facilitate characterization of cultivated hepatocytes.
